# Appendicitis in a Six-Month-Old Child With Coincidental Pneumonia

**DOI:** 10.7759/cureus.39429

**Published:** 2023-05-24

**Authors:** Seth J Deskins, Cassie Abbey, Amber Vozar, Richard Brant

**Affiliations:** 1 Internal Medicine-Pediatrics, West Virginia University School of Medicine, Morgantown, USA; 2 Pediatrics, West Virginia University School of Medicine, Morgantown, USA

**Keywords:** surgery, ileus, pediatrics, pneumonia, appendicitis

## Abstract

Appendicitis is an exceedingly uncommon diagnosis in infancy and, thus, is typically not considered a differential diagnosis for this population. Its atypical presentation, with a wide range of clinical manifestations, creates a diagnostic challenge for physicians. Along with this, a patient’s inability to articulate their pain adds another layer of diagnostic challenge. Here, we present the case of a six-month-old infant with a complicated hospital course of pneumonia and subsequent ileus, who was later found to have appendicitis with a surrounding abscess.

## Introduction

Appendicitis rarely manifests in infants, but when it does, the presentation is highly variable, ranging from abdominal distension to respiratory distress [1,2]. The classic presentation of appendicitis with periumbilical pain radiating to the right lower quadrant only presents in less than half of patients under 12 years of age and is nearly impossible to appreciate in a nonverbal child [1]. Appendicitis would typically be low on the differential list in the infant population, making diagnosis difficult. Here we discuss the case of a six-month-old male infant who presented initially with right upper lobe pneumonia with associated emesis that evolved into persistent abdominal distension, ileus, and the eventual diagnosis of appendicitis.

## Case presentation

**Figure 1:**
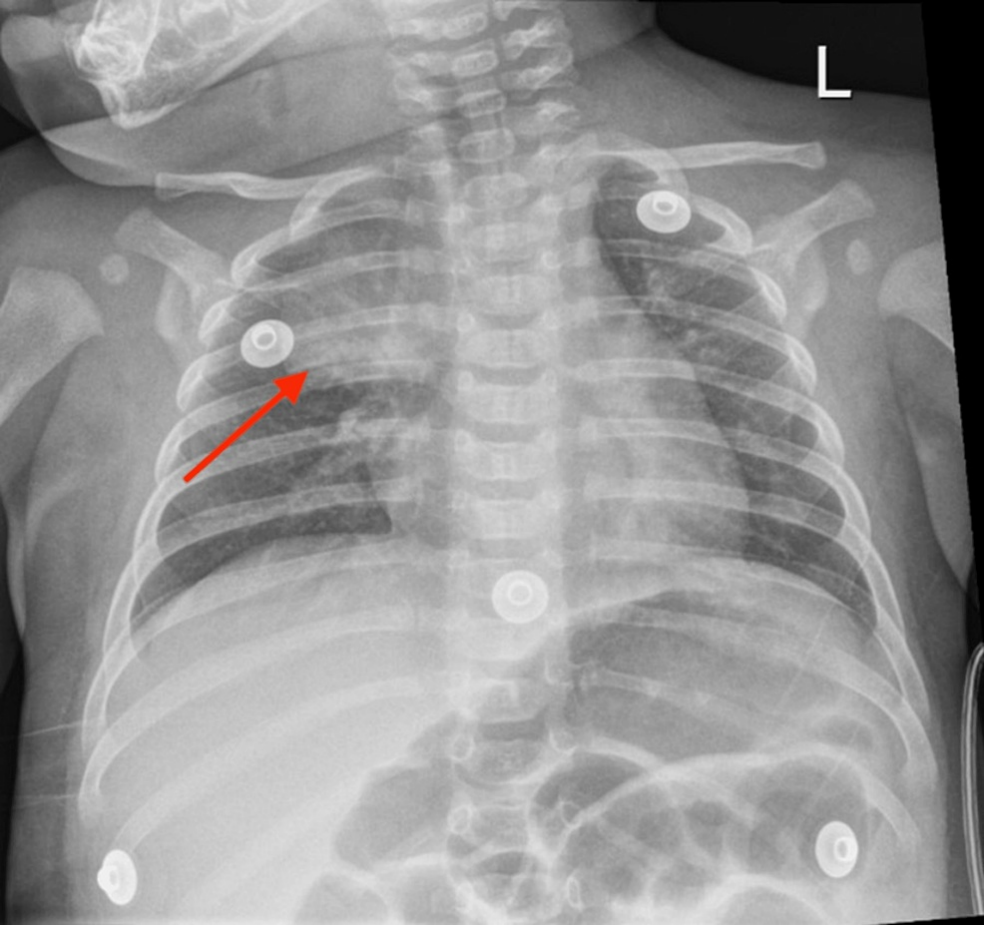
Chest radiograph showing focal right upper lobe pneumonia (arrow)

A six-month-old male presented to the emergency department with a one-day history of low-grade fever, diarrhea, and emesis. In the emergency department, he was febrile at 38.2 degrees Celsius, tachycardic at 198 beats/minute, and irritable on physical exam with coarse breath sounds at the right apex. Lab values revealed an elevated procalcitonin of 5.00 ng/mL (<0.50 ng/mL) and C-reactive protein of 67.6 mg/L (<8.0 mg/L). A chest radiography showed a right upper lobe opacity (Figure 1). 

Blood cultures were obtained and showed no growth for 72 hours. He received a 20 mL/kilogram normal saline fluid bolus, which improved his tachycardia, and was started on ampicillin-sulbactam parenterally. 

**Figure 2:**
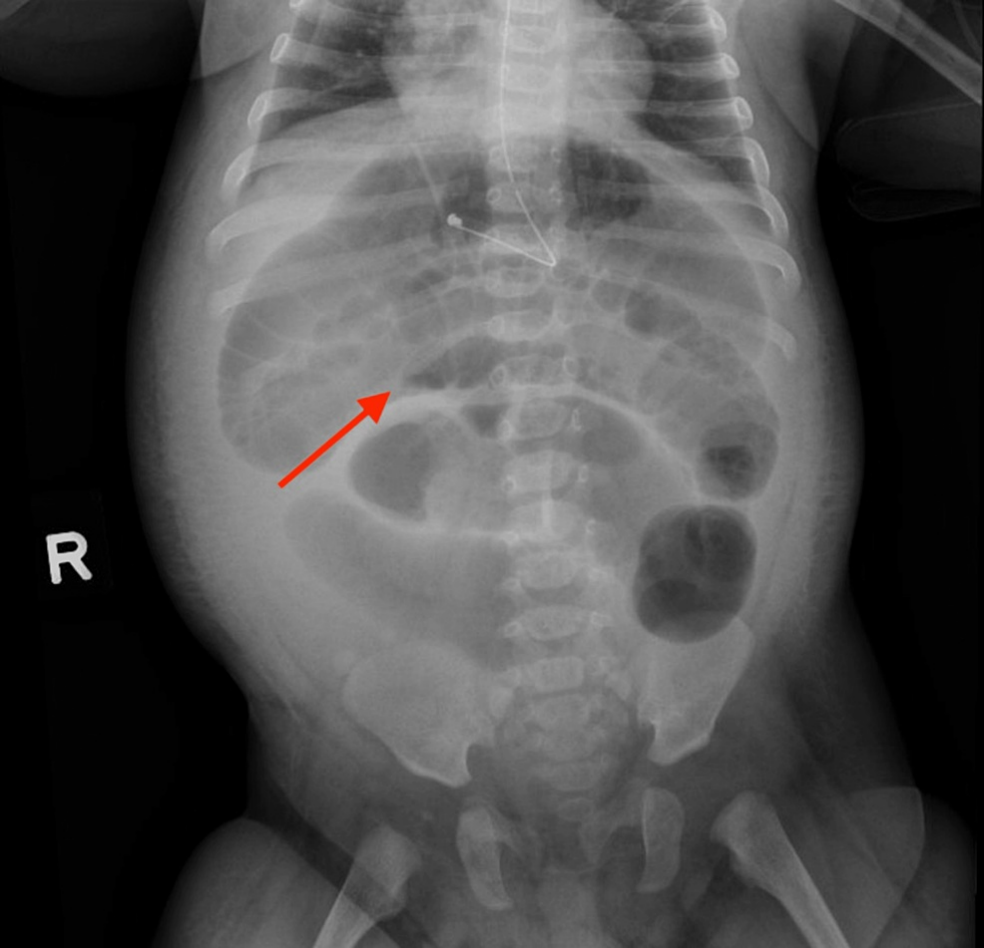
Abdominal radiograph showing marked dilated bowel loops (arrow)

The patient’s inflammatory markers improved while on antibiotics, but he continued to have decreased bowel movements and persistent emesis with oral feeds. Feeds were discontinued, and he was started on maintenance intravenous fluids. His physical exam evolved over the next few days to a firm non-distended abdomen with bowel sounds present. An abdominal radiograph was obtained and showed air-filled small and large bowel loops in the left abdomen, consistent with an ileus-type pattern (Figure 2). 

After approximately one day of bowel rest, re-feeding was attempted with an oral rehydration solution. He initially tolerated but over the span of approximately 12 hours, he had gradually worsening physical exam findings and abdominal radiographs. 

Pediatric Surgery was consulted and was concerned for a small bowel obstruction and recommended bowel decompression through a nasogastric tube. Even with this intervention, a repeat abdominal radiograph showed worsening dilated and tubular bowel loops. Further imaging with an abdominal ultrasound was then performed and was negative for appendicitis and did not show free fluid. Given a few days with unresolved ileus pattern, Pediatric Surgery recommended an exploratory endoscopic laparotomy. The procedure revealed a perforated appendix with a fecalith and a surrounding abscess with peritonitis that was causing a partial small bowel obstruction. The contents expressed from the abscess were sent for culture and yielded multiple Gram-positive rods, lactose-fermenting Gram-negative rods, and *Proteus* species. Based on these culture results, Pediatric Infectious Disease recommended changing antibiotics to metronidazole and ceftriaxone.

Postoperatively, the patient rapidly improved over the following day. Feeds were resumed on postoperative day 1 and gradually increased, and his bowel function resumed two days postoperatively. Intra-operative cultures ultimately speciated to vancomycin-resistant enterococcus (VRE), which was sensitive to linezolid. Therefore, the patient was discharged on parenteral linezolid and ciprofloxacin per infectious disease recommendations with close outpatient pediatric surgery follow-up. 

## Discussion

While relatively common in pediatric adolescent populations and adulthood, appendicitis is infrequently diagnosed in younger children, especially in infancy [1]. Appendicitis historically presents with anorexia, nausea, emesis, and right lower quadrant pain concerning a surgical abdomen. However, infants tend to present with fever, irritability, grunting, cough, rhinitis, lethargy, and abdominal distension or rigidity, with fever being the best predictor of appendicitis in this age group [1,2]. Although it is apparent that these symptoms, alone or in combination, could be explained by a myriad of other, even benign, illnesses, the combination of an inability to articulate and localize pain, a multitude of etiologies for abdominal discomfort and fever, and atypical presentations make appendicitis a challenging diagnosis for those in this age group. 

Up to 57% of appendicitis cases are missed or misdiagnosed in children under 12 years of age [1], which leads to increased morbidity and mortality. While a delay of four days or greater in diagnosis for patients under the age of two can lead to catastrophic outcomes, even a 36-hour delay increases the risk of perforation by up to 65% [1,2], as seen in our case report. In one study, children who were misdiagnosed displayed more frequent perforation and peritoneal abscess formation and were the only cases in which mortality occurred [2]. Anatomically, appendicitis is less likely to occur in those under one year of age. At this age, the appendix is funnel-shaped with a wider opening, generally preventing obstruction [3]. However, the omentum at this time is underdeveloped, which often leads to rapid peritonitis due to the inability to handle infection if perforation does occur [3]. When appendicitis does occur in neonates, the thinner appendix wall makes these patients especially susceptible to perforation, with 70% of patients aged less than three years developing perforation within 48 hours of symptom onset [4]. 

A crucial aspect of our case report is the initial presentation of pneumonia. Although the concurrent presentation of appendicitis and pneumonia was observed, it could be that the aspiration of vomitus secondary to appendicitis may have led to pneumonia given the presence of dilated bowel loops on initial imaging. It is also possible that, in this case, the treatment of pneumonia with ampicillin-sulbactam may have modified the course of this patient’s appendicitis and prevented clinical decompensation. In fact, it has been shown that antibiotics alone are an effective treatment for uncomplicated appendicitis in older children [5]. Nevertheless, the presentation of pneumonia marred the clinical picture and led to a delay in diagnosis. Given the significant consequences of delay or misdiagnosis of appendicitis in infants, we advocate that physicians and research-based clinical practice guidelines (e.g., scoring systems to assist with diagnosis such as the Alvarado Scoring System) need to maintain lower thresholds to rule out appendicitis.

## Conclusions

This case of a six-month-old male with pneumonia and appendicitis emphasizes the importance of maintaining a high clinical suspicion. Certain things are common in the differential diagnosis but serious intra-abdominal pathology such as infection and appendicitis must be ruled out. We encourage providers to rely on clinical examination as imaging may not yield the true underlying pathology, as in our case. Timely diagnosis is paramount, as complications tend to arise within the first 48 hours of symptom onset. The case presented here, although atypical, emphasizes a need for increased awareness of diagnosis and the management of appendicitis in infancy.
